# Lymphocyte count or percentage: which can better predict the prognosis of advanced cancer patients following palliative care?

**DOI:** 10.1186/s12885-017-3498-8

**Published:** 2017-08-02

**Authors:** Weiwei Zhao, Peng Wang, Huixun Jia, Menglei Chen, Xiaoli Gu, Minghui Liu, Zhe Zhang, Wenwu Cheng, Zhenyu Wu

**Affiliations:** 1 Department of Integrated Therapy, Fudan University Shanghai Cancer Center, Shanghai, China; 2 Department of Oncology, Shanghai Medical College, Fudan University, Shanghai, China; 3 Department of Integrative Oncology, Fudan University Shanghai Cancer Center, Shanghai, China; 4 Clinical Statistics Center, Fudan University Shanghai Cancer Center, Shanghai, China; 5 Department of Biostatistics, School of Public Health, Key Laboratory of Public Health Safety and Collaborative Innovation Center of Social Risks Governance in Health, Fudan University, Shanghai, China

**Keywords:** Lymphocyte count, Lymphocyte to white blood cell ratio, Prognostic value, Advanced cancer, Palliative care

## Abstract

**Background:**

The lymphocytes played an important role in the natural history of cancer. The aim of this study was to explore the prognostic value of lymphocyte count and percentage for survival in advanced cancer patients receiving palliative care.

**Methods:**

A retrospective review of clinicopathological data from 378 consecutive advanced cancer patients and 106 extended follow-up patients treated with palliative care was conducted. Kaplan–Meier curves and multivariate cox regression analyses were used to evaluate the relationships of peripheral lymphocyte count (LC) and lymphocyte to white blood cell ratio (LWR) with overall survival (OS).

**Results:**

The median values for pretreatment LC and LWR were 1.1 (IQR, 0.8 ~ 1.5 × 10^9^/L) and 0.138 (IQR, 0.086 ~ 0.208). The median survival times across LWR quartiles were 19, 47, 79, and 101 days (*P* < 0.001). Multivariate analysis indicated that patients in the highest quartiles of LC and LWR had an HR of 1.082 (95% CI 0.777 ~ 1.506, *P* = 0.642) and 0.466 (95% CI 0.328 ~ 0.661, *P* < 0.001), respectively, compared with patients in the lowest quartiles. Furthermore, only the dynamic changes of LWR were confirmed as an independent prognostic factor for overall survival during the follow-up (HR = 0.396, 95% CI 0.243 ~ 0.668; *P* = 0.001), as were primary tumor site and ECOG. No effect was observed for the dynamic changes of LC.

**Conclusions:**

Our findings demonstrate that measurement of the dynamic changes of LWR prior to treatment and during follow-up may represent a simple and new powerful prognostic factor for patients with advanced cancer, unlike measurement of LC. As a bedside marker of immune status, the prognostic role of LWR should be further evaluated in prospective studies.

**Electronic supplementary material:**

The online version of this article (doi:10.1186/s12885-017-3498-8) contains supplementary material, which is available to authorized users.

## Background

Cancer is a major public health problem and the epidemic is set to rise worldwide. It can lead to severe health consequences, especially advanced cancers, which pose great therapeutic challenges and usually cause death [1]. Given these issues, palliative care has been accepted as an essential component throughout the cancer trajectory [2]. Prognostication of life expectancy in advanced cancer patients is highly needed for clinicians in palliative care as identifying the likelihood of imminent death would assist physicians and facilitate clinical decision-making to help patients and their families prepare for the time ahead [3, 4]. However, there are no proven factors to aid in this prediction for patients with shorter life expectancies [5]. The Clinical Prediction of Survival, which is most commonly used for prognostication in palliative care, is subject to inherent non-reproducibility that limits its accuracy and clinical application [6].

The immune system is thought to play an important role in the natural history of cancer by influencing cancer development and progression [7]. Lymphocytes are essential effector cells during cancer immunosurveillance [8]. Low absolute lymphocyte count (LC), has been associated with inferior outcomes in various cancers, including lung cancer, breast cancer, colorectal cancer, ovarian cancer, renal cell cancer, pancreatic adenocarcinoma, and others [9–14]. Comparatively, the lymphocyte to white blood cell ratio (LWR) was less considered in previous studies and only found to have relationships with nasopharyngeal carcinoma, hepatocellular carcinoma, and colorectal carcinoma [15–17]. In addition, to our knowledge, no studies have investigated the prognostic value of lymphocytes in advanced cancer patients undergoing palliative care.

To explore this question, we investigated the prognostic value of not only the absolute counts but also the percentage of lymphocytes for overall survival in advanced cancer patients and identified the clinical significance of the changes in peripheral LC and LWR in advanced cancer patients after palliative care.

## Methods

### Data collection and study cohort

Consecutive inpatients treated at the palliative care unit of Fudan University Shanghai Cancer Center (FUSCC) in Shanghai, China between July 2013 and October 2015 were considered for the study (Additional file 1: Figure S1). Demographics (age and gender), medical history (comorbidities, smoking status, and family history), tumor-related factors (primary tumor site and tumor stage), nutritional status and physical status (Eastern Cooperative Oncology Group, ECOG score) were obtained from the medical records of the patients. White blood cell (WBC) count and its differential counts were performed 1-3 days before palliative care. The LWR was calculated as the absolute lymphocyte count divided by the total WBC count. An unintentional weight loss >5% in the previous 3 months or a food intake below 75% of the normal requirement in the preceding week were considered to be an abnormal nutritional status according to the ESPEN guidelines for nutrition screening [18]. The presence of a concomitant disease was defined as self-reported cardiac disease, hypertension, diabetes, or any cerebrovascular disease.

Two study cohorts were identified in our study and the criteria have been previously illustrated [19]. Patients with the following inclusion criteria were enrolled to cohort 1: (1) a hospitalization for palliative care; (2) the presence of various cancers confirmed by histopathology or at least cytology; (3) availability of pretreatment peripheral blood test results from 1 to 3 days prior to palliative care; and (4) availability of all clinical data. Patients with benign or early stage (I, II) tumors, and those with acute active infectious disease were excluded from the analysis. Patients in cohort 1 who had a second admission were enrolled to cohort 2. In cohort 1, the associations of several potential risk factors with overall survival (OS) were examined and qualified patients with full medical records were enrolled. In cohort 2, patients with further treatment were enrolled and the associations of changes in lymphocytes with OS were evaluated accordingly. The last follow-up date was in December 2015. This study was conducted in accordance with the ethical standards of the Declaration of Helsinki and was approved by the Ethics Committee of FUSCC. Informed consent was waived because of the retrospective nature of the study.

### Statistics analysis

Continuous data are presented as the mean ± standard deviation (SD) or median and interquartile range (*P*_*25*_ ~ *P*_*75*_), and comparisons were made by the Wilcoxon sum rank test or Kruskal-Wallis H test. Categorical data are described as totals and frequencies and comparisons were made by the chi-squared or Fisher’s exact test as appropriate. The Kaplan-Meier method was used to calculate the survival rates, and these were compared using the log-rank test. The correlations between lymphopenia, established prognostic factors, and overall survival rates were analyzed using the univariate Cox regression analysis. Important factors identified by the univariate analysis were selected as covariates to construct a multivariate proportional hazards model for survival. Given the variation in the optimal LC and LWR thresholds for different tumor types, the thresholds were not specified. Instead, the LCs and LWRs were stratified into quartiles. The median OS was calculated for each quartile and quartile-1 was used as the reference category for comparing OS. A two-sided *P* value less than 0.05 was considered statistically significant. Statistical analyses were performed using SAS 9.4 (Cary, NC, USA) and R software version 3.3.1 (Institute for Statistics and Mathematics, Vienna, Austria).

## Results

### Patient characteristics

Cohort 1 was comprised of 378 qualified patients and 106 of those with readmission data were grouped into cohort 2. The median duration of follow-up for patients in cohort 1 and cohort 2 was 445 days (range, 1 ~ 882 days) and 509 days (range, 28 ~ 882 days), respectively.

In cohort 1, there were about 10% more males than females, and only a few patients had stage III (6.08%) disease. The three most frequent tumors in our study were gastrointestinal (52.38%), thoracic (22.75%), and urogenital (15.61%) tumors. Approximately one thirds of patients had a family history of cancer, a history of smoking and a normal nutritional status; two fifths of patients had concomitant disease and were in poor physical condition (ECOG > 3). LC and LWR were not statistically significant in most of the clinicopathological features. Nutritional status was the only feature that was significantly associated with both LC and LWR (Table 1).

**Table 1:** Comparisons of baseline LC and LWR values and clinicopathological features in cohort 1 (*N* = 378)

Clinicopathological features	*n*	LC *M* (*P*_25_ ~ *P*_75_)	*P*	LWR *M* (*P*_25_ ~ *P*_75_)	*P*
Age (*M* (*P*_25_ ~ *P*_75_))	64(56 ~ 73)	--		--	
Gender			0.751		0.019
Male	209(55.29%)	0.8(1.1 ~ 1.5)		0.081(0.133 ~ 0.194)	
Female	169(44.71%)	0.8(1.1 ~ 1.5)		0.095(0.145 ~ 0.222)	
Tumor stage			0.586		0.058
III	23(6.08%)	0.9(1.2 ~ 1.5)		0.127(0.190 ~ 0.214)	
IV	355(93.92%)	0.8(1.1 ~ 1.5)		0.084(0.136 ~ 0.207)	
Primary tumor site			0.110		0.308
Gastrointestinal tumors	198(52.38%)	0.7(1 ~ 1.4)		0.081(0.131 ~ 0.190)	
Thoracic cancer	86(22.75%)	0.9(1.2 ~ 1.7)		0.092(0.142 ~ 0.213)	
Urogenital neoplasms	59(15.61%)	0.8(1.1 ~ 1.6)		0.088(0.147 ~ 0.235)	
Head and neck neoplasm	16(4.23%)	0.8(1.1 ~ 1.4)		0.089(0.137 ~ 0.246)	
Other tumors	19(5.03%)	0.9(1.3 ~ 1.6)		0.099(0.171 ~ 0.245)	
Family history			0.935		0.999
No	264(70.78%)	0.8(1.1 ~ 1.5)		0.083(0.141 ~ 0.203)	
Yes	109(29.22%)	0.8(1.1 ~ 1.5)		0.091(0.127 ~ 0.222)	
Unknown	5				
Smoking history			0.478		0.005
No	265(71.62%)	0.8(1.1 ~ 1.5)		0.092(0.145 ~ 0.214)	
Yes	105(28.38%)	0.8(1 ~ 1.5)		0.075(0.118 ~ 0.185)	
Unknown	8				
ECOG			0.564		<0.001
< 3	218(57.67%)	0.8(1.1 ~ 1.5)		0.094(0.146 ~ 0.231)	
> =3	160(42.33%)	0.8(1.1 ~ 1.5)		0.072(0.113 ~ 0.173)	
Concomitant disease			0.440		0.968
No	229(60.58%)	0.8(1.1 ~ 1.5)		0.085(0.138 ~ 0.214)	
Yes	149(39.42%)	0.8(1.1 ~ 1.6)		0.088(0.139 ~ 0.198)	
Nutrient status			0.012		<0.001
Normal	107(28.50%)	0.8(1.2 ~ 1.6)		0.108(0.169 ~ 0.233)	
Abnormal	268(71.50%)	0.8(1 ~ 1.45)		0.079(0.129 ~ 0.196)	
Unknown	3				

*LC* lymphocyte count; *LWR* lymphocyte to white blood cell ratio

In cohort 2, changes in LCs and LWRs were calculated according to the records at baseline and their last visit. A patient was assigned to the decreased subgroup if the patient had a change < 0, otherwise, the patient was assigned to the increased subgroup. Clinicopathological features were distributed similarly to those in cohort 1, and almost all factors were balanced except for tumor stage (Table 2). As we expected for LC and LWR, patients in stage III were a larger proportion of the increased than patients in stage IV.

**Table 2:** Clinicopathological features of the patients in cohort 2 (*N* = 106)

Clinicopathological features	*n*	LC		*P* value	LWR		*P* value
		Decreased	Increased		Decreased	Increased	
Age (*M* (*P*_25_ ~ *P*_75_))	106	64(56-69)	62(53-72)	0.3708	64(56-72)	62(53-70)	0.2784
Gender				0.0517			0.9756
Male	56(52.83%)	23(43.40%)	33(62.26%)		36(52.94%)	20(52.63%)	
Female	50(47.17%)	30(56.60%)	20(37.74%)		32(47.06%)	18(47.37%)	
Tumor stage				0.0382			0.0010
III	13(12.26%)	3(5.66%)	10(18.87%)		3(4.41%)	10(26.32%)	
IV	93(87.74%)	50(94.34%)	43(81.13%)		65(95.59%)	28(73.68%)	
Primary tumor site				0.6794			0.3937
Gastrointestinal tumors	60(56.60%)	33(62.26%)	27(50.94%)		41(60.29%)	19(50.00%)	
Thoracic ancer	14(13.21%)	6(11.32%)	8(15.09%)		9(13.24%)	5(13.16%)	
Urogenital neoplasms	23(21.70%)	9(16.98%)	14(26.42%)		11(16.18%)	12(31.58%)	
Head and neck neoplasm	6(5.66%)	3(5.66%)	3(5.66%)		5(7.35%)	1(2.63%)	
Other tumors	3(2.83%)	2(3.77%)	1(1.89%)		2(2.94%)	1(2.63%)	
Family history				0.2943			0.4234
No	73(68.87%)	34(64.15%)	39(73.58%)		45(66.18%)	28(73.68%)	
Yes	33(31.13%)	19(35.85%)	14(26.42%)		23(33.82%)	10(26.32%)	
Smoking history				0.0905			0.8157
No	74(69.81%)	41(77.36%)	33(62.26%)		48(70.59%)	26(68.42%)	
Yes	32(30.19%)	12(22.64%)	20(37.74%)		20(29.41%)	12(31.58%)	
ECOG				0.1093			0.6462
< 3	81(76.42%)	37(69.81%)	44(83.02%)		51(75.00%)	30(78.95%)	
> =3	25(23.58%)	16(30.19%)	9(16.98%)		17(25.00%)	8(21.05%)	
Concomitant disease				0.5529			0.8093
No	63(59.43%)	33(62.26%)	30(56.60%)		41(60.29%)	22(57.89%)	
Yes	43(40.57%)	20(37.74%)	23(43.40%)		27(39.71%)	16(42.11%)	
Nutrient status				0.9360			0.4560
Normal	42(40.00%)	21(40.38%)	21(39.62%)		25(37.31%)	17(44.74%)	
Abnormal	63(60.00%)	31(59.62%)	32(60.38%)		42(62.69%)	21(55.26%)	
Unknown	1	1	-		1	-	

*SD* standard deviation, *LC* lymphocyte count, *LWR* lymphocyte to white blood cell ratio

### Prognostic value of LWR

**Fig. 1:**
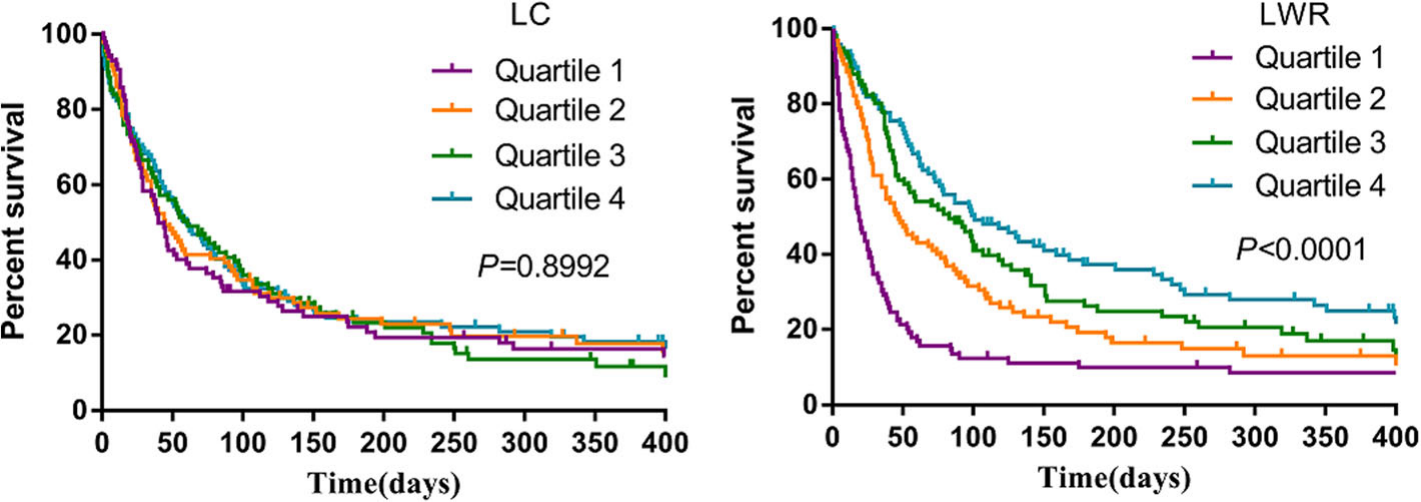
Overall survival of patients with palliative care stratified by pretreatment LC and LWR (cohort 1). LC: lymphocyte count; LWR: lymphocyte to white blood cell ratio

To evaluate the prognostic value of the LC and LWR, the hazard ratio (HR) was calculated for each quartile, with quartile 1 being used as a reference. For patients categorized by quartile of LC, the median survival time was 40 days (95% CI 32.1 ~ 48.0) for quartile 1, 45 days (95% CI 28.2 ~ 61.8) for quartile 2 (HR = 1.135, *P* = 0.471), 58 days (95% CI 31.8 ~ 84.2) for quartile 3 (HR = 1.081, *P* = 0.655), and 60 days (95% CI 40.5 ~ 79.5) for quartile 4 (HR = 1.082, *P* = 0.642). For patients categorized by quartile of LC, the median survival time was 40, 45, 58 and 60 days, respectively. Similarly, if the patients were categorized by quartile of LWR, the median survival time was 19, 47, 79 and 101 days, respectively (Table 3 and Fig. 1). LC was not a significant prognostic factor, while LWR was a significant prognostic factor. Our analysis revealed that the higher the LWR, the better the survival.

**Table 3:** Multivariate Cox regression analysis for LC and LWR in cohort 1(*N* = 378)

Prognostic factors	Median survival time (95% CI)	HR^a^(95% CI)	*P* value
LC			0.912
Quartile 1(<0.8 × 10*9/L)	40.0 (32.1 ~ 48.0)	Reference	-
Quartile 2(~1.1 × 10*9/L)	45.0 (28.2 ~ 61.8)	1.135(0.805 ~ 1.600)	0.471
Quartile 3(~1.5 × 10*9/L)	58.0 (31.8 ~ 84.2)	1.081(0.768 ~ 1.520)	0.655
Quartile 4(≥1.5 × 10*9/L)	60.0 (40.5 ~ 79.5)	1.082(0.777 ~ 1.506)	0.642
LWR			
Quartile 1(<0.086)	19.0(13.0 ~ 25.0)	Reference	-
Quartile 2(~0.138)	47.0(30.8 ~ 63.2)	0.563(0.407 ~ 0.778)	0.001
Quartile 3(~0.208)	79.0(40.3 ~ 117.7)	0.532(0.381 ~ 0.742)	<0.001
Quartile 4(≥0.208)	101.0(55.1 ~ 146.9)	0.466(0.328 ~ 0.661)	<0.001

*LC* lymphocyte count, *LWR* lymphocyte to white blood cell ratio, *OS* overall survival, *HR* hazard ratio, *CI* confidence interval

^a^Cox regression model controlling for Age; Gender; Family history; Smoke history; Nutrient status; ECOG; Primary tumor site; Tumor stage; Concomitant disease

### Association of LC, LWR, and the changes in these values with OS

**Fig. 2:**
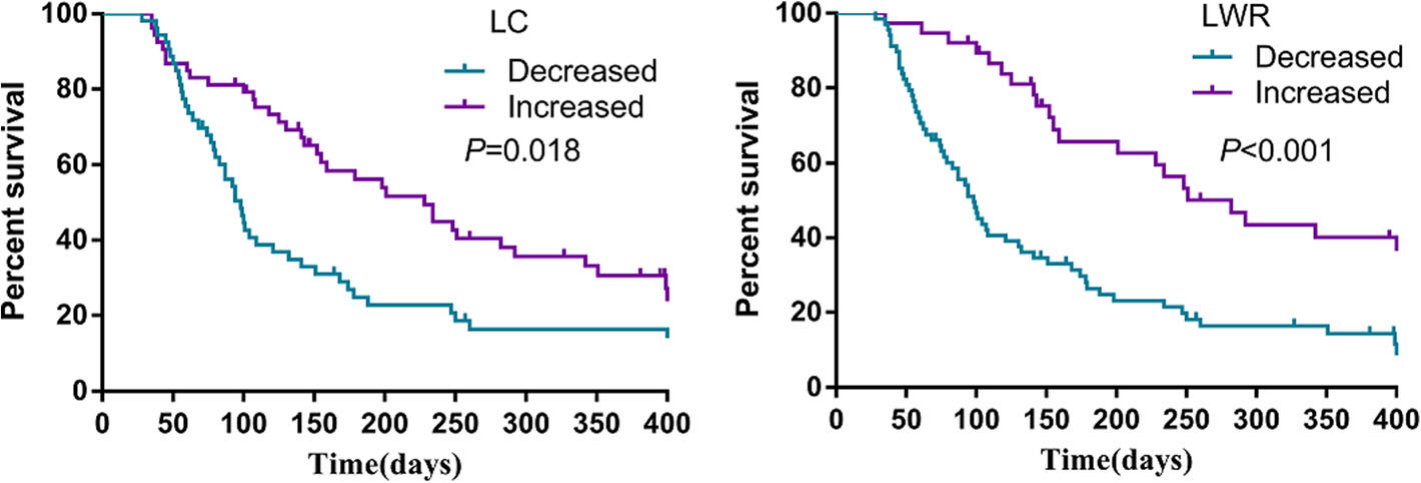
Overall survival of patients with palliative care stratified by LC and LWR changes (cohort2). LC: lymphocyte count; LWR: lymphocyte to white blood cell ratio

To validate the prognostic significance of the dynamic changes in LC and LWR, we studied cohort 2 in which patients were divided into increased and decreased subgroups based on changes in the LC or LWR. Patients with an increased LC or LWR had a significantly longer survival time than those with a decreased LC or LWR [228 days (158.7 ~ 297.3) vs. 98 days (82.8 ~ 113.2), *P* = 0.018; and 282 days (205.5 ~ 358.5) vs. 98 days (81.1 ~ 114.9), *P* < 0.001] (Fig. 2). However, multivariate analyses showed that an increase in LC was not associated with OS (HR: 0.673, 95% CI: 0.419 ~ 1.081; *P* = 0.101), while a decrease in LWR was significantly related to a poor OS (HR: 0.396, 95% CI: 0.243 ~ 0.668; *P* = 0.001), as were primary tumor site and ECOG (Table 4).

**Table 4:** Adjusted HRs for overall survival stratified by LC and LWR changes in cohort 2 (*N* = 106)

Clinicopathological features	LC Model		LWR Model	
	Adjusted HR(95% CI)	*P* value	Adjusted HR (95% CI)	*P* value
Gender (female vs. male)	0.859(0.496 ~ 1.486)	0.586	0.847(0.492 ~ 1.460)	0.551
Age	1.006(0.985 ~ 1.028)	0.565	0.999(0.979 ~ 1.020)	0.921
Primary tumor site	0.787(0.618 ~ 1.003)	0.053	0.751(0.590 ~ 0.955)	0.019
Tumor stage (IV vs. III)	2.813(1.076 ~ 7.356)	0.035	1.971(0.738 ~ 5.263)	0.176
Family history (Yes vs. No)	1.405(0.846 ~ 2.333)	0.189	1.424 (0.862 ~ 2.351)	0.167
Smoke history (Yes vs. No)	0.897(0.491 ~ 1.639)	0.724	0.716(0.390 ~ 1.314)	0.280
Nutrient (Abnormal vs. Normal)	0.987(0.591 ~ 1.649)	0.961	0.983(0.597 ~ 1.617)	0.945
ECOG	1.614(1.140 ~ 2.283)	0.007	1.850(1.291 ~ 2.650)	0.001
Concomitant disease (Yes vs. No)	1.165(0.714 ~ 1.899)	0.542	1.278(0.785 ~ 2.082)	0.324
LC (Increased vs. Decreased)	0.673(0.419 ~ 1.081)	0.101	--	--
LWR(Increased vs. Decreased)	--	--	0.396(0.243 ~ 0.668)	0.001

*LC* lymphocyte count, *LWR* lymphocyte to white blood cell ratio, *HR* hazard ratio, *CI* confidence interval

## Discussion

Lymphocytes are a non-specific yet commonly used bedside marker of host immunity. Interestingly, the results of the present single-center retrospective study showed that advanced cancer patients with low LWRs had a significantly shorter OS. In addition, decreased LWR was demonstrated to be an independent prognostic factor for poor outcome in patients with advanced cancer following palliative care. However, these results were not found when analyzing LC. Therefore, this study shows that measurement of LWR prior to treatment and during follow-up may be a novel and effective prognostic factor for advanced cancer patients in a palliative setting. This merits further investigation and potentially enables the ability to predict end-of-life patients with advanced cancer.

The level of lymphocytes in the blood is most commonly obtained as a component of a complete blood cell count with differential. It is easy and inexpensive to detect the lymphocyte level this way and is already in use as part of the pretreatment workup at FUSCC. In recent years, mounting evidence has demonstrated that the LC is an independent prognostic marker in various cancers, such as lung cancer, breast cancer, colorectal cancer, ovarian cancer, renal cell cancer, pancreatic adenocarcinoma, and others [9–14]. The LWR has been less investigated in cancers and has only been found to have relationships with nasopharyngeal carcinoma, hepatocellular carcinoma, and colorectal carcinoma [15–17]. Up to now, no study has investigated the relationship between LC or LWR and outcomes of advanced cancer patients in a palliative setting. Therefore, LC and LWR were evaluated with OS in advanced cancer patients in the present study. Patients with abnormal nutrient status were found to have significantly lower LCs and LWRs, which is in accordance with data from Ota Y et al. [20], who found that nutrient deprivation suppressed the proliferation of peripheral blood lymphocytes. In addition, the LWR was lower in male patients and in patients with a smoking history or poor ECOG, while these differences were not found with LC. Taken together, these results suggest that a low level of lymphocytes may reflect a poor health status and poor prognosis in advanced cancer patients. Next, we evaluated the efficacy of peripheral blood lymphocytes for predicting OS in advanced cancer patients. The Kaplan-Meier survival curves demonstrated that patients with low LWR exhibited significantly shorter survival times according to the LWR quartiles (*P* < 0.001). Furthermore, the multivariate analysis revealed that patients with a high LWR appeared to have significantly decreased hazards of death compared to those with a low LWR. The HRs were substantially lower for adverse outcomes in the highest, third, and second LWR quartiles compared with those in the lowest quartile. However, the association between LWR as a surrogate for immune function and prognosis was not found for LC. The prognostic significance of LC and LWR discordance is less clear. The possible reason may be that LC levels may vary in the same patient from day-to-day and are thus not static [21]. Taylor JM et al. reported that the CD4 lymphocyte percentage possessed greater prognostic significance to predict the development of AIDS and had less diurnal variability than CD4 lymphocyte count [22]. Previous studies have not found a significant association between LC and cancer survival, which might be due to a neglect of the discordance. Thus, further research is warranted to verify these conflicting results yielded by LC and LWR and their effects on patient survival.

Given the intrinsic variability in lymphocyte levels, we evaluated the dynamic changes of LC and LWR after palliative care. Elevations of both LC and LWR mainly occurred in stage III compared to stage IV. Patients with increased LC or LWR had significantly prolonged survival time compared to patients with decreased LWR. However, only decreased LWR was identified as a significant and strong independent poor predictive factor for OS especially after adjusting for confounding factors (primary tumor site and ECOG). In the case of LWR and survival in advanced cancer, we theorize that patients who have an elevated LWR after palliative care may have a more robust immune reaction, which may convey an improved prognosis. In a word, LWR and its dynamic changes could be applied in the clinical practice not only because they are convenient and easy-measured, but also independent prognostic factors to OS. The popularization of these indexes would help the doctors to make medical decisions without any more harm on patients and wasting the medical resources. They provide additional information in predicting OS, which would assistant the patients and their families with proper hospice care. Specifically, patients with high LWR could receive some active treatment to extend the survival, while for patients with low LWR, unnecessary toxic therapies might be avoided and timely hospice care might be a better choice.

The mechanism underlying the association between low LWR and poor cancer prognosis is not well understood. However, it is likely multifactorial and remains to be fully elucidated. Latest studies have explored the complicated interactions between tumor and host immune cells and the corresponding immune response. The composition of the immune microenvironment in tumors includes various immune cell types, including lymphocytes, dendritic cells, macrophages, mast cells, neutrophils, and myeloid-derived suppressor cells [23]. Lymphocytes constitute two-thirds of immune cells, and approximately 80% are T cells, which aid in combating tumors. T cells distinguish from other lymphocytes such as B cells and natural killer cells, they have T cell receptors on the cell surface. Cytotoxic T cells, which are known as CD8+ T cells, destroy tumor cells through binding to antigen presented by MHC class I molecules [24]. These cytotoxic CD8+ T cells are activated by the combination of antigen presentation and co-stimulatory signals (CD80/CD86) [25]. T helper cells (Th cells), which are also known as CD4+ T cells, play a central role in orchestrating the immune response to cancers. They help maturation of B cells into plasma cells and memory B cells, activate CD8+ T cells and macrophages [24]. Th cells are activated through peptide antigens expressed by MHC class II molecules on the surface of antigen-presenting cells. When Th cells are activated, they undergo rapid division into various types such as Th1, Th2, Th3, Th17, Th9, or tumor-infiltrated follicular helper and release different cytokines to promote various active immune reactions. In the past few years, cancer immunotherapy, such as new immune modulators of cytokines and blockers of cytotoxic T-lymphocyte-associated protein 4 and programmed cell death protein 1/programmed death-ligand 1, have emerged as safe and effective alternatives for the treatment of cancers that do not respond to classical treatments [25]. In these scenarios, a low peripheral lymphocyte level may indicate a poor lymphocyte-mediated immune response to tumors and suggest poor prognosis.

The results of this study should be interpreted in the context of several inherent limitations. First, a main drawback is that this study is derived from a single tertiary care center with a retrospective design and a relatively small sample size. Second, as lymphocyte subsets were not routinely measured in standard clinical practice, the types of lymphocytes specifically associated with the survival of advanced cancer patients were not determined. Third, the infection of virus and the injury of radiation, which may potentially affect the lymphocytes, were not taken into account in the study. Despite these limitations, this is the first study to assess the relationship between peripheral blood lymphocytes and the mortality of advanced cancer patients to determine if it can be frequently used as a bedside immunosuppressive indicator of this clinical state. Therefore, larger and more detailed prospective studies are needed to further elucidate these relationships.

## Conclusions

In conclusion, our study is the first to demonstrate that measurement of the dynamic changes of LWR prior to treatment and during follow-up is associated with prognosis of advanced cancer patients. LWR rather than LC can be better utilized in this clinical setting. As a bedside barometer of host immune function, LWR can help to provide critical information for physicians to determine optimal time management for patients and their families in the palliative care context. Future investigations are warranted to confirm these preliminary findings.

## Additional file

Additional file 1: Figure S1. Flow chart of patients excluded from the study. (DOCX 28 kb)

## Abbreviations

CI: Confident interval
ECOG: Eastern Cooperative Oncology Group
FUSCC: Fudan University Shanghai Cancer Center
HR: Hazard ratio
LC: Lymphocyte count
LWR: Lymphocyte to white blood cell ratio
OS: Overall survival
SD: Standard deviation
WBC: White blood cell
