# Supplementary material for: Lymphocyte count or percentage: which can better predict the prognosis of advanced cancer patients following palliative care?
Source: BMC Cancer. 2017 Aug 2;17:514. doi: 10.1186/s12885-017-3498-8 (PMC5541405; doi:10.1186/s12885-017-3498-8)
Supplement: Additional file 1: Figure S1. — Flow chart of patients excluded from the study. (DOCX 28 kb) [file 12885_2017_3498_MOESM1_ESM.docx]

The flow chart of the patients enrolled is listed as follows:

A total of 405 patients were retrieved from FUSCC

Excluded benign patients (*n*=2)

Malignant patients (*n*=403)

Excluded Stage I to II (*n*=18)

Advanced cancer patients (*n*=385)

Excluded patients with active infectious disease (*n*=7)

Eligible to be involved in **cohort 1** (*n*=378)

**Figure S1 Flow chart of patients excluded from the study.**
